# The Role of Irisin/FNDC5 Expression and Its Serum Level in Breast Cancer

**DOI:** 10.3390/ijms24108628

**Published:** 2023-05-11

**Authors:** Kamil Cebulski, Aleksandra Piotrowska, Alicja Kmiecik, Katarzyna Haczkiewicz-Leśniak, Urszula Ciesielska, Jędrzej Grzegrzółka, Karolina Jabłońska, Hanna Romanowicz, Beata Smolarz, Piotr Dzięgiel, Marzenna Podhorska-Okołów, Katarzyna Nowińska

**Affiliations:** 1 Division of Histology and Embryology, Department of Human Morphology and Embryology, Wroclaw Medical University, 50-368 Wroclaw, Poland; kamil.cebulski@umw.edu.pl (K.C.); jedrzej.grzegrzolka@umw.edu.pl (J.G.);; 2 Division of Ultrastructural Research, Wroclaw Medical University, 50-368 Wroclaw, Poland; 3 Department of Pathology, Polish Mother Memorial Hospital-Research Institute, 93-338 Lodz, Poland; 4 Department of Human Biology, Faculty of Physiotherapy, Wroclaw University of Health and Sport Sciences, 51-612 Wroclaw, Poland

**Keywords:** irisin, FNDC5, EMT, E-cadherin, SNAIL, SLUG, TWIST, breast cancer (BC)

## Abstract

Irisin (Ir) is an adipomyokine formed from fibronectin type III domain-containing protein 5 (FNDC5), which can be found in various cancer tissues. Additionally, FNDC5/Ir is suspected of inhibiting the epithelial-mesenchymal transition (EMT) process. This relationship has been poorly studied for breast cancer (BC). The ultrastructural cellular localizations of FNDC5/Ir were examined in BC tissues and BC cell lines. Furthermore, we compared serum levels of Ir with FNDC5/Ir expression in BC tissues. The aim of this study was to examine the levels of EMT markers, such as E-cadherin, N-cadherin, SNAIL, SLUG, and TWIST, and to compare their expression levels with FNDC5/Ir in BC tissues. Tissue microarrays with 541 BC samples were used to perform immunohistochemical reactions. Serum levels of Ir were assessed in 77 BC patients. We investigated FNDC5/Ir expression and ultrastructural localization in MCF-7, MDA-MB-231, and MDA-MB-468 BC cell lines and in the normal breast cell line (Me16c), which was used as the control. FNDC5/Ir was present in BC cell cytoplasm and tumor fibroblasts. FNDC5/Ir expression levels in BC cell lines were higher compared to those in the normal breast cell line. Serum Ir levels did not correlate with FNDC5/Ir expression in BC tissues but were associated with lymph node metastasis (N) and histological grade (G). We found that FNDC5/Ir correlated moderately with E-cadherin and SNAIL. Higher Ir serum level is associated with lymph node metastasis and increased grade of malignancy. FNDC5/Ir expression is associated with E-cadherin expression level.

## 1. Introduction

Breast cancer (BC) is the most prevalent cancer in women, and it is considered the most common cause of death in cancer patients [1]. Treatment of patients with BC, particularly in the advanced stages of the disease, remains a challenge. Therefore, it seems essential to search for new treatment methods, further exploration of the pathomechanism of this cancer, and the detection of new diagnostic markers. Our previous study showed a potential protective effect of FNDC5/irisin (FNDC5/Ir) in BC. We observed a negative correlation between the expression level of FNDC5/Ir and the presence of metastases. In addition, higher expression levels were associated with longer patient survival [2]. Due to the unclear causes of the relationships that we previously reported, we decided to continue the research.

Irisin (Ir) is a transmembrane protein [3] that was first observed in the muscle fibers of mice [4]. The authors found that under the influence of physical activity, there was an increased level of FNDC5 in muscle fibers and its further transformation into a new protein known as Ir. Next, it is secreted outside the cell and, as a hormone, affects metabolic changes in adipose tissue, which results in increased thermogenesis [4]. Expression of FNDC5/Ir has been confirmed in many other tissues, including adipose, kidney, liver, cardiomyocytes, skin, and cerebellum. It is formed as a result of the cleavage of 112 amino acids of the FNDC5 chain [5]. Next, Ir undergoes glycosylation, which is one of the most important processes of post-translational modification and significantly changes its physicochemical properties. Inhibition of glycosylation leads to decreased Ir secretion. The molecular mass of the non-glycosylated form is about 13kD and about 20kD in the case of the glycosylated form [6]. Apart from physiological conditions, increased expression of Ir was also observed in various types of cancers, e.g., in breast, prostate, digestive tract (including liver), bone, lung, and thyroid cancer [7,8]. It is believed that elevated levels of Ir in tumor tissue led to the inhibition of cell division and mitochondrial ATP synthesis [9].

It was also suggested that Ir inhibited the proliferation, migration, and EMT of cancer cells by affecting the PI3K/AKT/SNAIL pathway [10]. Such relationships were observed by Shao et al. [11] in an in vitro study on lung cancer cells. In the same study, Ir also correlated negatively with the expression level of SNAIL, which is involved in EMT. The inhibitory effect of Ir on cell proliferation (including breast, lung, bone, and prostate cancer cells) was confirmed in many other in vitro studies [10,12,13,14,15]. On the other hand, some papers did not confirm the effect of Ir on the inhibition of proliferation, adhesion, or colony formation by cancer cells [16]. Stimulation of cell invasion was also observed in liver cancer through the PI3K/AKT pathway [17]. In turn, an increased level of FNDC5/Ir expression in cancer-associated fibroblasts (CAFs) in patients with NSCLC was a negative prognostic factor [5]. CAFs participate in cancer progression by stimulating angiogenesis and EMT [18]. In vivo studies showed that Ir serum levels were decreased in various types of cancers (e.g., prostate, osteosarcoma, bladder, breast, colorectal, liver, gastric cancers) [19]. It is suspected that serum Ir levels can be used as a biomarker of different types of cancer [19].

EMT is a process during which an epithelial cell (expression of E-cadherin) transforms into a mesenchymal cell (expression of N-cadherin). The cell loses structural connections with other cells and loses its polarity. E-cadherin is one of the transmembrane proteins and also one of the most significant epithelial markers. It participates in the formation of the cytoskeleton and is responsible for maintaining normal cell polarity [20]. It plays a crucial role in maintaining intercellular connections and is necessary to preserve the stability of cell membranes. The decrease in its expression level is associated with EMT [21]. In turn, N-cadherin is one of the markers detected in mesenchymal cells. It serves as an EMT marker and is associated with the progression of many cancers. EMT is found in physiological processes (wound healing) and tumor progression. Physiological processes are strictly controlled. However, uncontrolled EMT occurs during tumor progression [22]. SNAIL, SLUG, and TWIST are considered the most important transcription factors involved in EMT. SNAIL-induced EMT leads to a decrease in the level of E-cadherin in cells and an increase in the expression levels of N-cadherin, vimentin, and fibronectin [22]. SNAIL is one of the transcription factors. Together with HDAC1, HDAC2, and mSin3A, it forms a complex that affects the downregulation of the expression of E-cadherin. SNAIL expression is controlled by many pathways, including PI3K/AKT, MAPK, Shh Notch, Wnt, and TGH-β. The increase in the expression level of SNAIL is associated with the promotion of EMT [23]. Ir is suspected of inhibiting SNAIL expression and function by inhibiting the PI3K/AKT pathway [11] and STAT3 [10]. SLUG also belongs to the SNAIL protein family and has similar properties to SNAIL [24]. It can form complexes with another protein known as TWIST. This combination inhibits the expression of many proteins, including the expression of E-cadherin. Importantly, such a complex also acts as a transcription factor for SLUG. Knockdown of SLUG completely blocks the ability of TWIST to suppress E-cadherin transcription and thus induces EMT [25]. Increased expression levels of SLUG, SNAIL, and TWIST are transcription factors that promote EMT transition [22].

The relationships between the level of Ir expression, its secretion, EMT, and cancer progression are very complex and may result in opposite effects under different conditions. Therefore, further research is warranted to explain the differences in such relationships. In this study, the level and localization of the above markers in the tissues of BC patients were determined. These markers have not yet been compared with the level of FNDC5/Ir expression in such a large cohort. We also examined the localization and expression level of FNDC5/Ir in cell lines. Furthermore, we compared serum Ir levels with FNDC5/Ir expression levels in BC tissue. To the best of our knowledge, it has never been reported before. Moreover, the ultrastructural cellular localization of Ir in BC tissues was assessed for the first time. Additionally, due to the differences in the research results related to the effect of Ir on the progression of cancer in different types, the aim of this study was to examine the levels of EMT markers, such as E-cadherin, N-cadherin, SNAIL, SLUG, and TWIST, and to compare their expression levels with the level of FNDC5/Ir expression in BC.

## 2. Results

### 2.1. The Association of FNDC5/Ir with Epithelial-to-Mesenchymal (EMT) Markers

**Figure 1:**
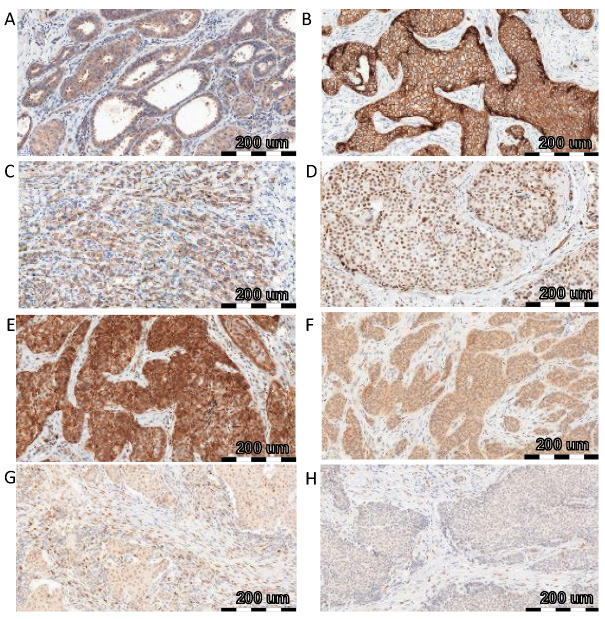
Comparison of FNDC5/Ir ((**A**)—IRS score 8) expression with E-cadherin ((**B**)—IRS score 12), N-cadherin ((**C**)—IRS score 8), SNAIL ((**D**)—IRS score 3 and % of nuclear expression 4, (**E**)—IRS score 12 and % of nuclear expression 4), SLUG ((**F**)—IRS score 8 and % of nuclear expression 3) and TWIST ((**G**)—IRS score 4 and % of nuclear expression 2, (**H**)—IRS score 4 and % of nuclear expression 2) using immunohistochemistry (IHC) (positive reactions—brown cell cytoplasm) in breast cancer (BC), magnification ×200.

To investigate the relationship between FNDC5/Ir expression level and EMT transition, correlations with EMT markers, such as E-cadherin and N-cadherin, SNAIL, SLUG, and TWIST, were evaluated. We compared the previously obtained results of FNDC5/Ir expression in BC tissues [2] with the level of expression of the above markers. Table 1 shows the characteristics of BC patients with the division into high and low Ir expression levels according to the median (table obtained from Cebulski et al., 2022 [2]). FNDC5/Ir expression in BC patient tissues was observed in the cytoplasm of tumor cells (Figure 1A), while E-cadherin showed membrane expression (Figure 1B). In turn, the expression of N-cadherin was noted in the cytoplasm (Figure 1C). For the transcription factors SNAIL (Figure 1D,E), SLUG (Figure 1F), and TWIST (Figure 1G,H) in BC tumors, we observed cytoplasmic and nuclear expression. Therefore, the levels of cytoplasmic and nuclear expression of each protein were evaluated.

**Figure 2:**
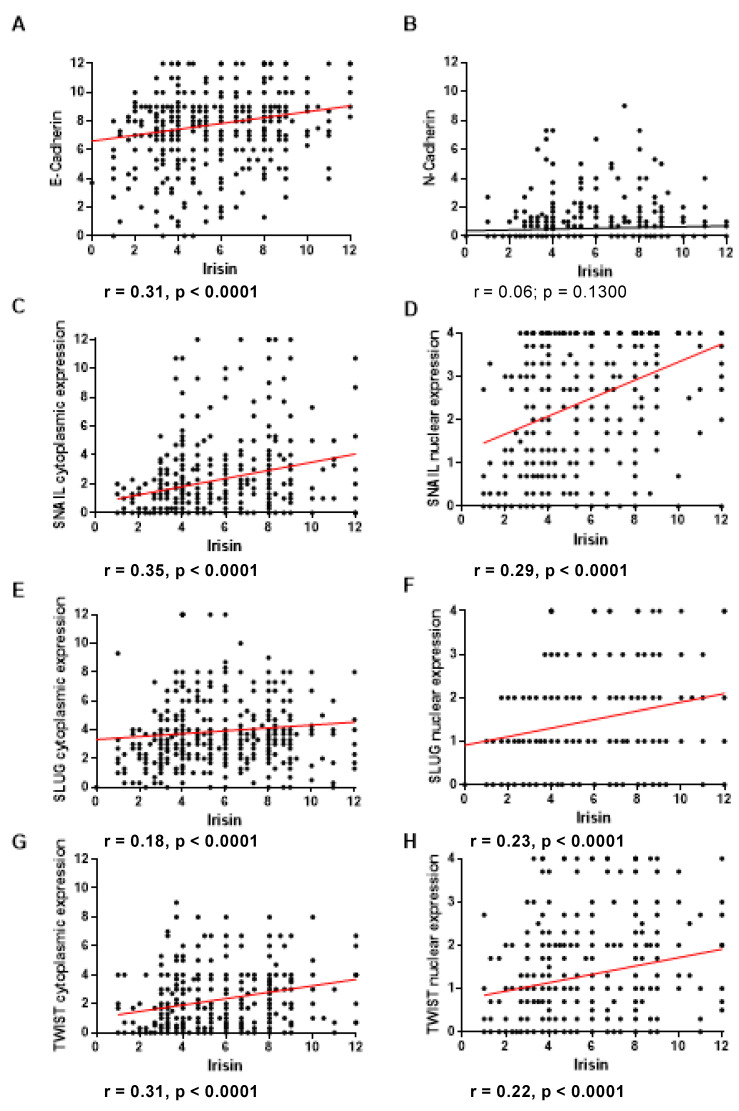
Correlation of FNDC5/Ir expression level with E-cadherin (**A**) and N-cadherin (**B**), cytoplasmic (**C**) and nuclear (**D**) SNAIL expression levels, cytoplasmic (**E**) and nuclear (**F**) SLUG expression levels, cytoplasmic (**G**) and nuclear (**H**) TWIST expression levels in breast cancer (BC) (sample size *n* = 541).

For each protein, we analyzed the correlation of its expression level with the level of FNDC5/Ir in tumor cells. E-cadherin showed a moderate positive correlation with the FNDC5/Ir expression level (r = 0.31, *p* < 0.0001) (Figure 2A). The expression level of N-cadherin was not related to FNDC5/Ir expression (Figure 2B). Cytoplasmic and nuclear SNAIL expression correlated moderately positively with FNDC5/Ir expression level (r = 0.35; *p* < 0.0001; r = 0.29, *p* < 0.0001, respectively) (Figure 2C,D). In contrast, in the case of the transcription factor SLUG, we observed a weak positive correlation with the expression level of FNDC5/Ir for cytoplasmic and nuclear expression (r = 0.18, *p* < 0.0001; r = 0.23, *p* < 0.0001, respectively) (Figure 2E,F). We observed a positive moderate correlation for cytoplasmic expression of TWIST (r = 0.31; *p* < 0.0001) and a weak positive correlation for nuclear (r = 0.22; *p* < 0.0001) expression of TWIST (Figure 2G,H).

### 2.2. Comparison of Ir Levels in Tissues and Serum of Breast Cancer Patients

Serum Ir concentrations were evaluated by ELISA. The association of the results of serum Ir levels in patients with the clinicopathological data, as well as with the results of the FNDC5/Ir expression level in the BC tissue were investigated.

**Figure 3:**
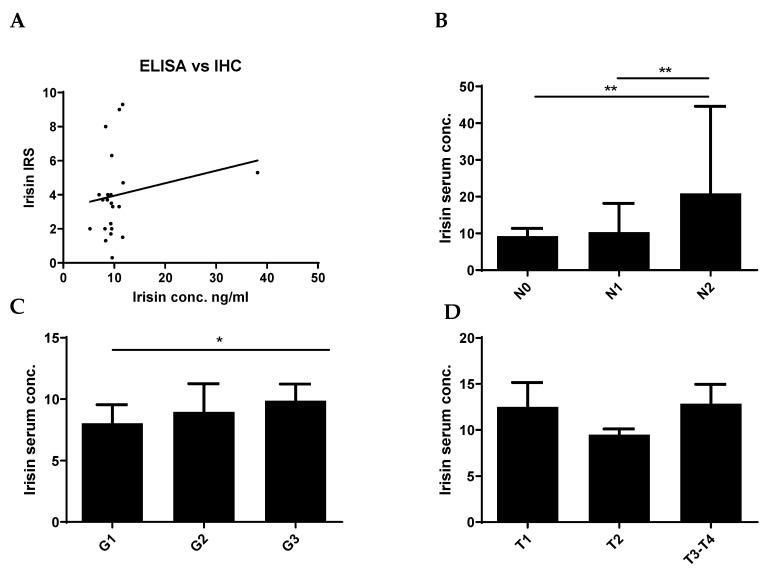
Comparison of serum Ir concentrations in breast cancer (BC) patients with expression levels in BC tissue (**A**). Comparison of Ir concentrations in BC patients according to the lymph node status [N] (**B**), histological grade [G] (**C**), and tumor size [T] (**D**), * *p* < 0.05 ** *p* < 0.01 (sample size *n* = 77). The lymph node status, histological grade, and tumor size were evaluated based on the World Health Organization Criteria and the 8th TNM edition.

We did not observe an association between the expression level of FNDC5/Ir in BC tissue examined by IHC and serum concentrations of Ir (Figure 3A). However, we observed higher serum concentrations of Ir in patients with a large number of lymph node metastases (N2, mean 20.86 ± 23.7 SD) compared to patients without metastases (N0, mean 9.25 ± 2.1 SD) and with single lymph node metastases (N1, mean 10.33 ± 7.7 SD) (Mann-Whitney U test; *p* = 0.0055, *p* = 0.0072, respectively) (Figure 3B). We observed higher concentrations of Ir in N1 patients compared to N0 subjects. However, the difference was not statistically significant. In addition, serum concentrations of Ir were higher with the increase in tumor malignancy (mean G1—8.02 ± 1.4 SD; G2—8.96 ± 2.2 SD; G3—9.86 ± 1.4 SD). We observed higher serum Ir concentrations in patients with G3 compared to G1 (*p* = 0.0166) (Figure 3C). We found no statistically significant differences in Ir levels for the increasing tumor size (Figure 3D). Table 2 shows the characteristics of BC patients with the division into low and high serum Ir concentration levels according to the median. The analysis with the Chi^2^ test showed a difference between the groups with high and low Ir expression (division according to the median value, Table 2) only in different age groups of patients (≤60 and >60 years of age). Table 3 shows the associations of Ir serum level with the clinicopathological characteristics in patients with BC.

### 2.3. FNDC5/Ir Expression in Breast Cancer Cell Lines

Some examinations were conducted on BC cell lines (MCF-7, MDA-MB-231, MDA-MB-468). The normal breast cell line (Me16c) was used as the control. We estimated the FNDC5/Ir expression level in cells by measuring the level of immunofluorescence using confocal microscopy. In addition, we examined the expression level of *FNDC5* mRNA in cells using RT-PCR.

**Figure 4:**
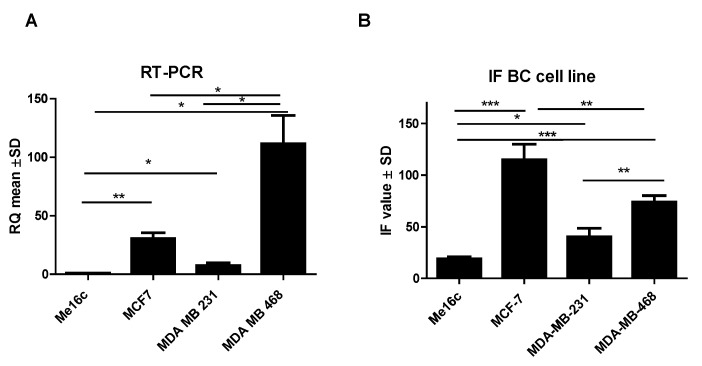
Comparison of mRNA FNDC5 expression levels detected by RT-PCR (**A**) and FNDC5/Ir levels (**B**) in the normal breast cell line (Me16c) and different types of BC cell lines (MCF-7, MDA-MB-231, MDA-MB-468) * *p* < 0.05 ** *p* < 0.01 *** *p* < 0.001.

*FNDC5* mRNA expression levels in normal breast cells (Me16c) were significantly lower (mean 1.03 ± 0.05 SD) compared to the levels found in the cells of all BC lines (MCF-7, mean 30.71 ± 8.43 SD, *p* = 0.0037; MDA-MB-231, mean 7.58 ± 4.05 SD, *p* = 0.0492; MDA-MB-468, mean 111.60 ± 41.93 SD, *p* = 0.0103). In addition, the expression levels were significantly different between BC cell lines. The highest level of expression of the *FNDC5* gene was observed in MDA-MB-468 cells. The difference between the *FNDC5* mRNA levels in MCF-7 and MDA-MB-231 lines was statistically significant (*p* = 0.0306; *p* = 0.0129, respectively) (Figure 4A).

**Figure 5:**
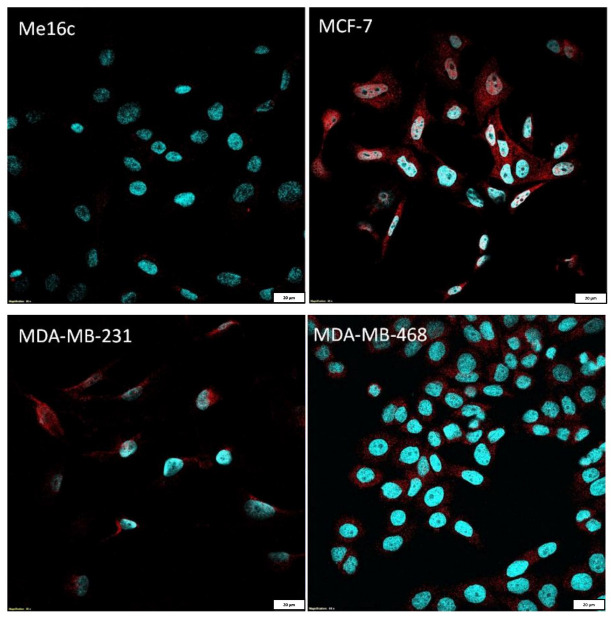
Comparison of FNDC5/Ir expression by confocal microscopy in the normal breast cell line (Me16c) and different BC cell lines (MCF-7, MDA-MB-231, MDA-MB-468).

Additionally, the measurement of immunofluorescence (IF) showed that the level of FNDC5/Ir expression in normal breast cells (Me16c) was significantly lower (mean 18.54 ± 4.50 SD) compared to the level in the cells of all BC lines (MCF-7—mean 125.60 ± 36.63 SD, *p* < 0.0001; MDA-MB-231 mean 37.81 ± 20.47 SD, *p* = 0.0327; MDA-MB-468 mean 74.13 ± 15.40 SD, *p* < 0.0001). The difference between the FNDC5/Ir levels in MDA-MB-231 and MCF-7, MDA-MB-468 lines was statistically significant (*p* = 0.0014; *p* = 0.0094, respectively) (Figure 4B). FNDC5/Ir expression by confocal microscopy in the normal breast cell line (Me16c) and different BC cell lines are given in Figure 5.

### 2.4. Ultrastructural Localization of Ir in Breast Cancer Cells

**Figure 6:**
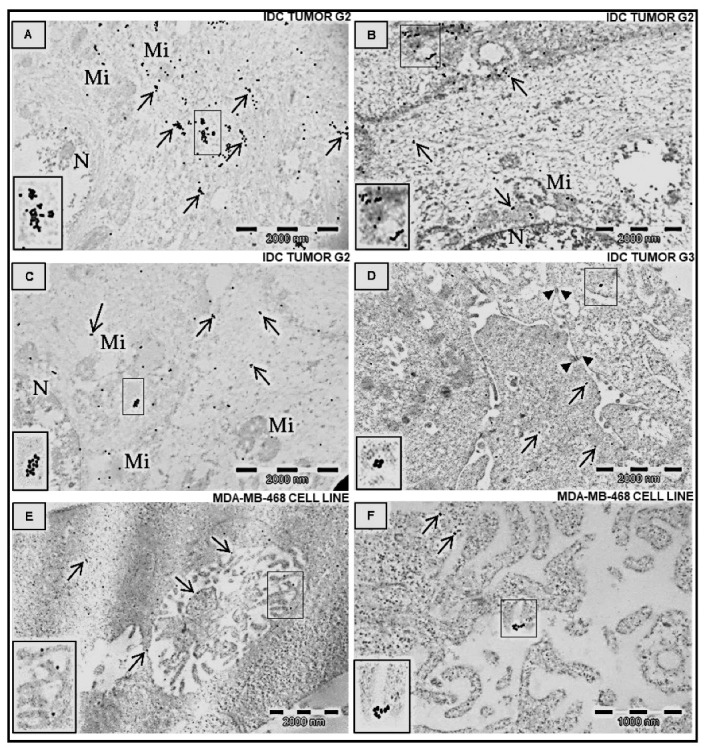
Immunolocalization of Ir in transmission electron microscopy. Ultrathin section examination of human adenocarcinoma BC cells (from tumors, (**A**–**D**) and MDA-MB 468 cell line, (**E**,**F**)). The specific primary antibody against FNDC5/Ir was applied. Next, the ultrathin sections were labeled with the secondary antibody conjugated with the 20 nm-colloidal gold nanoparticles, which shows the antigen distribution in the cells. Arrows indicate positive gold nanoparticles. A strong reaction was detected in the cytoplasm of cancer cells, in the mitochondria, and at the border of the cell membranes of neighboring cells. Note the localization of Ir near the specific microvilli–like structure (**E**) and at the cytoplasmatic processes of BC cells (**F**). Brief double staining with UranyLess solution and lead citrate (3%). IDC—invasive ductal carcinoma at different grades of malignancy, N—nucleus, Mi—mitochondrion.

**Figure 7:**
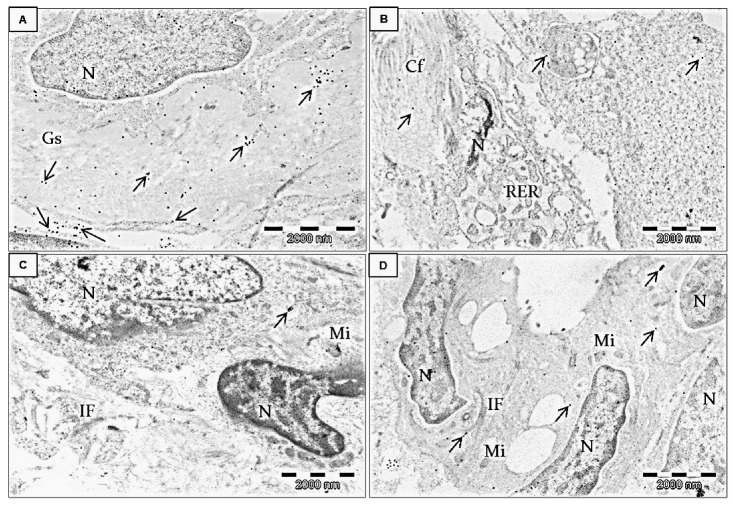
Immunolocalization of Ir in transmission electron microscopy. Ultrathin section examination of human adenocarcinoma BC cells from the breast tumor microenvironment (stroma). All electronograms show invasive ductal carcinoma G2. The specific primary antibody against FNDC5/Ir was applied as previously described, followed by applying the species-specific secondary antibody conjugated with 20 nm-colloidal gold nanoparticles. Arrows indicate positive gold nanoparticles. Strong immunogold reaction was detected in the extracellular matrix and cancer-associated fibroblasts (in the cytoplasm and at the border of the cell membrane). Brief double staining with UranyLess solution and lead citrate (3%). N—nucleus, Gs—ground substance of the extracellular matrix (**A**), Cf—collagen fibers, RER—rough endoplasmic reticulum in the fibroblast (**B**). IF—intermediate filaments (**C**), Mi—mitochondrion (**D**).

Immunolocalization of FNDC5/Ir at the ultrastructural level was performed after the application of the post-embedding method. First observations were made on invasive ductal carcinoma (IDC) tumors at different stages of malignancy (G2 and G3) (Figure 6A–D and Figure 7). In both stages, Ir was visible in the tumor cells, the extracellular matrix, and stromal cells, particularly in fibroblasts (Figure 7). Fibroblasts were recognizable by accompanying collagen, elastic fibers, and extracellular fluid. These cells were elongated, with the abundant, slightly dilated rough endoplasmic reticulum, which suggests an active release of proteins and stacks of Golgi cisternae. However, in BC cells the strongest immunogold reaction was found in the mitochondria, cell membranes of the neighboring cells, near the intermediate filaments, and the rough endoplasmic reticulum. Moreover, we also found some inflammatory cells and many capillaries in the stroma of breast tumors. We also analyzed MDA-MB-468 BC cell lines embedded in the form of cell pellets entrapped in the fibrin clot and post-stained with osmium tetroxide (Figure 6E,F). The most striking features were microvilli-like structures formed by the neighboring cancer cells and long cytoplasmic extensions. Colloidal gold nanoparticles were detected in these structures or the cytoplasm.

## 3. Discussion

This study is a continuation of our previous studies evaluating the expression level of FNDC5/Ir in BC tissue [2]. In this study, we assessed the association of serum levels of Ir with clinicopathological factors and IHC tissue expression, ultrastructural localization of FNDC5/Ir expression, and their correlation with EMT markers in a group of 541 BC patients.

For metastasis to occur, the EMT process must occur in the cancer cells [26]. The most important change is the disappearance of E-cadherin expression and the appearance of N-cadherin, which is characteristic of the mesenchymal phenotype [21,27]. In our study, the level of FNDC5/Ir expression positively correlated with the level of E-cadherin expression. However, we did not notice a relationship between FNDC5/Ir levels and N-cadherin expression. The results suggest that FNDC5/Ir may be associated only with the pathway of E-cadherin expression. Retaining a high level of E-cadherin expression in cancer cells maintains the epithelial phenotype of the cell and blocks EMT progression. We found a positive correlation between FNDC5/Ir and E-cadherin expression levels, which is consistent with other studies. In their research on lung cancer cell lines, Shao et al. [11] showed that cell exposure to Ir caused an increase in the level of E-cadherin expression. Similarly, Zhu et al. [28] observed an increase in E-cadherin expression after the administration of Ir to ovarian cancer cells (SKOV3 and A2780). They also observed that the amount of phosphorylated AKT decreased when Ir was administered to cells. This suggests that Ir influences EMT suppression of ovarian and pancreatic cancer cells through the PI3/AKT pathway by inhibiting AKT phosphorylation [28,29]. However, in the case of osteosarcoma, FNDC5/Ir inhibited STAT3 phosphorylation in the STAT3/SNAIL signaling pathway, which is also associated with the EMT process [10,11,15,28]. Our finding of a positive correlation between FNDC5/Ir and E-cadherin is consistent with previous studies indicating its inhibitory effect on EMT. This is also confirmed by the results of our earlier study on the level of FNDC5/Ir expression in BC tissues [2]. The higher the FNDC5/Ir expression level in BC cells, the lower the risk of metastases and the longer the patient s survival. These findings are consistent with the positive correlation between FNDC5/Ir and E-cadherin. Perhaps the lack of a relationship with N-cadherin indicates that Ir affects EMT only by increasing the expression of E-cadherin. On the other hand, Hollestelle et al. [30] observed in a large group of BC cell lines that the loss of E-cadherin expression was not necessary for cell reshaping to spindle shape and EMT. In both BC cell lines and tissue material, they observed that the loss of E-cadherin expression in cells that underwent EMT was not necessary. Moreover, the loss of E-cadherin was demonstrated in only half of metaplastic BC. Hollestelle et al. investigated whether the CpG1 and CpG3 hypermethylation of the E-cadherin gene in BC cell lines had an impact on EMT. They indicated that CpG3 hypermethylation was a prerequisite for the complete loss of E-cadherin. Interestingly, re-upregulation of E-cadherin expression did not restore the cells to their typical epithelial shape. Multiple BC cell lines showed high E-cadherin expression with the simultaneous expression of EMT markers. In addition, Hollestelle et al. [30] indicated that the expression of E-cadherin gene repressors, such as *SNAI1*, *SNAI2*, and *TWIST1*, was also observed in non-spindle cell lines. Additionally, the positive correlation between FNDC5/Ir and E-cadherin did not preclude the occurrence of EMT in BC cells, which is in line with the findings of Hollestelle et al. This indicates a high complexity of interactions between E-cadherin repressors.

In our study, we observed weak and moderate positive correlations between SLUG, TWIST, SNAIL, and FNDC5/Ir levels. Such correlations were found in the cytoplasm and the nucleus of BC cells. SLUG, TWIST, and SNAIL are transcription factors that induce EMT in the cell by modifying the expression of many proteins, including the inhibition of E-cadherin expression [22]. The positive correlations of SNAIL, SLUG, and TWIST transcription factors with FNDC5/Ir seem to contradict the positive correlation between E-cadherin expression and FNDC5/Ir. However, these correlations were weak and positive for SLUG and nuclear TWIST. On the other hand, a moderate correlation between SNAIL and FNDC5/Ir was observed for cytoplasmic and nuclear expression of this transcription factor and also between TWIST and FNDC5/Ir for cytoplasmic expression. This may indicate that higher FNDC5/Ir concentrations affect EMT in a more complex manner.

FNDC5/Ir may not only influence the levels of the transcription factors (SNAIL, SLUG, and TWIST) in the tissue but also their activity in the cell. Wang et al. [31] indicated that the expression, transport, and function of SNAIL were controlled in the cells also at the stage of post-translational modification by β-TrCP-induced ubiquitination and GSK3β-dependent phosphorylation. SNAIL in the cytoplasm is rapidly degraded. In turn, SNAIL in the nucleus is characterized by greater stability and is functionally active [31]. SNAIL phosphorylated by GSK3β is transported from the nucleus to the cell cytoplasm, where after ubiquitination by β-TrCP it is degraded inside the peroxisomes [31]. In our study, we observed a moderate correlation between FNDC5/Ir and SNAIL for cytoplasmic and nuclear expression. FNDC5/Ir could lead to a lack of SNAIL activity and could promote SNAIL transportation to the cytoplasm, but IHC only indicates a relationship and is not sufficient enough to prove it. Further studies are warranted to clarify the functional relationship of these proteins and whether FNDC5/Ir may affect the activity of the SNAIL transcription factor.

The high complexity of the effect of FNDC5/Ir on EMT was demonstrated by various studies [5,11,16,17]. Although most studies showed the inhibitory effect of Ir on the proliferation and migration of BC cells [10,12,13,14,15], some research did not confirm such findings. Moon et al. [16] did not observe a significant effect of Ir on the proliferation and migration of endometrial, colorectal, thyroid, and esophageal cancer cell lines. In the case of hepatocellular carcinoma, Shi et al. [17] found that FNDC5/Ir had a positive effect on the proliferation and invasion of cancer cells also by affecting the PI3K/Akt signaling pathway. Perhaps, the differences in the influence of Ir on EMT are specific to different types of cancer and are the result of the influence of Ir on particular metabolic pathways.

In our study, we also analyzed and compared serum and tumor levels of Ir from BC patients. We noticed a tendency for a positive relationship between the expression level of FNDC5/Ir in BC tissue and serum concentrations of Ir in patients. However, this relationship was not statistically significant. The lack of statistical significance may result from various reasons. First, newly synthesized FNDC5 in the cell must undergo a complex pathway before it is converted to glycosylated Ir and released into plasma. The release of Ir into the serum is still not fully understood. The factors affecting changes in plasma concentrations of Ir are still not clear. Such factors may include gender, age, the level of physical activity, or the stage of the menstrual cycle [32]. In addition, Ir can be secreted into the plasma by various tissues [3,4]. The complexity of the whole process indicates that plasma Ir concentrations do not necessarily correlate with the levels found in cancer cells. In our study, we used a relatively small group to compare Ir levels in serum and tissue.

However, when the relationship between plasma Ir levels and the clinicopathological data was analyzed, we observed significantly higher concentrations of Ir in patients with many lymph node metastases (N2) compared to patients without metastases (N0). So far, several studies on evaluating plasma concentrations of Ir in BC patients have been conducted. However, only two of them investigated the relationship between serum levels of Ir and the clinicopathological data of the patients. Our results are consistent with those presented by Provatopoulou et al. [33]. They also assessed plasma Ir levels in BC patients and showed that Ir levels were higher in patients with lymph node metastases, distant metastases, or high stages of cancer. However, Zhang et al. [34] examined Ir levels in the plasma of patients with BC without distant metastases and with spinal metastases. They found that high Ir levels in the serum were associated with the absence of spinal metastases. In our study, we did not check the relationship between Ir levels and distant metastases. However, Zhang et al. focused only on the presence or absence of distant metastases. They did not include comparisons of Ir levels with the clinical stage of the disease, lymph node metastases, or tumor size. The increase in plasma concentrations of Ir may occur in patients in advanced stages when the destruction of the body is observed. The increase in Ir expression in the N2 group may be related to the further progression of neoplastic disease. The literature suggests that in patients in more advanced stages of cancer, Ir is involved in developing cachexia, thus accelerating the destruction of the patient’s body [9].

In the immunogold study, we confirmed that FNDC5/Ir was present in the cytoplasm of BC cells in tissue as well as in cell lines. FNDC5/Ir was observed in the mitochondria, cell membranes, near the intermediate filaments, and the rough endoplasmic reticulum. This confirms FNDC5/Ir expression observed by IHC and IF. Additionally, we observed Ir in the extracellular matrix and the cells of stromal fibroblasts. Nowinska et al. [35] also detected Ir in the mitochondria, endoplasmic reticulum, and cytoplasm in the ultrastructure of A549, NCI-H522, and NCI-H1703 lung cancer cells. In addition, Ir has previously been observed in NSCLC stromal fibroblasts [5].

We also conducted research on BC cell lines. We examined the FNDC5 expression level and the FNDC5/Ir protein level. Studies on an in vitro model confirmed a higher level of FNDC5/Ir expression in BC tissues compared to NMBD tissues, which we previously observed [2]. As in the tissue material, we detected significantly lower FNDC5 mRNA expression and FNDC5/Ir expression in normal Me16c cells compared to MCF-7, MDA-MB-231, and MDA-MB-468 cells. These findings were also confirmed by studies on other types of cancer. Similarly, Pinkowska et al. [6] observed a higher level of FNDC5/Ir in HEp-2 laryngeal carcinoma lines compared to normal cells. Higher levels of Ir were also detected in colon cancer [36] and lung cancer lines compared to normal cells [5]. Differences in FNDC5/Ir levels might be the result of metabolic changes in BC cells and their progesterone (PR), estrogen (ER), and HER receptor status. We observed the highest expression of mRNA *FNDC5* in the MDA-MB-468 cell line which is the most aggressive of the tested cell lines because of the triple-negative receptor status (HER-, ER-, PR-). Cancer cells show accelerated metabolism associated with the intensification of glycolysis or mitochondrial biogenesis [37]. The above processes result in changes in the expression of many proteins, including peroxisome proliferator-activated receptor gamma coactivator 1 alpha (PGC1α) [38]. PGC1α is a transcription factor that controls the expression of many proteins, including FNDC5 [4]. The increased level of FNDC5/Ir expression in cancer cells may result from the increasing energy demand of the cancer cell and the modification of its metabolic processes. They lead to the intensification of glycolysis and obtaining energy mainly in the anaerobic mechanism. This process is commonly observed in cancer [39].

In conclusion, the expression level of FNDC5/Ir in BC cell lines was higher than in normal BC epithelial cells. Ultrastructural studies showed the presence of FNDC5/Ir in the cytoplasmic structures of BC tumor cells and stromal fibroblasts. Higher Ir serum levels are associated with lymph node metastasis and a higher grade of malignancy. However, serum Ir levels in patients did not reflect its levels in BC tumor cells. Serum Ir levels in patients might also be affected by its secretion by other tissues, e.g., muscle and adipose. Further research is warranted to clarify the mechanisms of Ir release from cells to plasma and the regulation of this process. Additionally, FNDC5/Ir expression is associated with the expression level of E-cadherin and correlates with EMT marker expression in BC tissues. However, the results are contradictory and need to be confirmed in further studies. Therefore, further studies are warranted to explain the mechanism by which FNDC5/Ir influences E-cadherin upregulation and EMT transition.

## 4. Materials and Methods

### 4.1. Patient Cohort

The study was conducted on archival material consisting of 541 tumor tissues (patient characteristics in Table 1) and 77 serum samples from BC patients. As the control, 61 cases of NMBD were used. The material was collected from January 2004 to December 2012. All BC patients were diagnosed and treated at the Polish Mother’s Memorial Health Institute in Łódź. The control samples of NMBD were obtained from the 4th Military Teaching Hospital in Wroclaw. The study was approved by the Bioethics Committee of Wroclaw Medical University (No. 726/2019 and KB-731/2019). The mean age of patients during the treatment period was 56 years (24–86 years). The treatment and follow-up were based on the World Health Organization criteria and the 8th TNM edition [40]. The histological grade (G) and the clinical stage of BC patients were determined.

### 4.2. Cell Line Culture

Molecular biology studies were conducted using the adherent BC cell lines (MCF-7, MDA-MB-231, MDA-MB-468) obtained from The American Type Culture Collection (Manassas, VA, USA). The Me16c cell line was used as the control. MCF-7 cells were cultured in an EMEM medium (Lonza, Basel, Switzerland). Leibovitz’s L-15 Medium (Sigma-Aldrich, St. Louis, MO, USA) was used to culture MDA-MB-231 and MDA-MB-468. Me16c was cultured in MEBM medium (Lonza, Basel, Switzerland) supplemented with insulin, hydrocortisone, bovine pituitary extract (BPE), and the human epidermal growth factor (hEGF). All media were supplemented with 10% fetal bovine serum (FBS) (Merck, Darmstadt, Germany), 1% penicillin/streptomycin (Merck, Darmstadt, Germany), and L-glutamine (Merck). The HERA cell incubator (Heraeus, Hanau, Germany) was used to maintain constant cell culture conditions (37 °C, 5% CO_2_ concentration, and a 95% humidity level).

### 4.3. Immunohistochemistry (IHC) on Tissue Microarrays (TMAs)

Tissue microarrays (TMAs) were performed on 541 BC and 61 NMBD sections. Slides with the whole BC or NMBD tissue sections were stained with hematoxylin and eosin and scanned with the Pannoramic Midi II (3D HISTECH Ltd., Budapest, Hungary) histological scanner. Three demonstrative sites with cancer were selected by the Pannoramic Viewer (3D HISTECH Ltd., RRID: SCR_014424, Budapest, Hungary). Subsequently, the selected cancer sites were transferred with a core of 1.5 mm to the tissue arrays using the TMA Grand Master (3D HISTECH Ltd.).

Immunohistochemical reactions were performed on each TMA section. Deparaffinization, hydration, and thermal epitope demasking were completed using a low pH Target Retrieval Solution (Agilent Technologies, Santa Clara, CA, USA) for 20 min at 97 °C in a Dako PT Link (Dako, Glostrup, Denmark) apparatus. The expression of proteins was detected by specific primary antibodies, i.e., polyclonal rabbit: anti-irisin/FNDC5 (dilution 1:50; code no. NBP2-14024; Novus Biologicals, Littleton, CO, USA) and anti-SNAIL (1:400, Clone, code 13099-1-AP, Proteintech, Rosemont, IL, USA), monoclonal mouse: anti-E-cadherin antibody (ready to use, Clone NCH-38, code IR059; Dako, Glostrup, Denmark), anti-N-cadherin (1:50, Clone 6G11, code M3613; Dako), anti-SLUG (1:50, clone A-7, sc-166476, Santa Cruz Biotechnology, Santa Cruz, CA, USA), and monoclonal mouse anti-TWIST (dilution 1:50, clone Twist2C1a, code ab-50887, Abcam, Cambridge, UK). DAKO Autostainer Link48 (Dako) automated system and an EnVision FLEX kit (Dako) were used to visualize the antigens.

At least two independent pathologists-researchers conducted the evaluation of the IHC reactions at ×200 magnification. The assessment was performed using a BX41 light microscope (Olympus, Tokyo, Japan) coupled with a visual circuit and the Cell D (Olympus) software. The nuclear expression levels of SNAIL, TWIST, and SLUG were determined using a five-point evaluation scale (% of nuclear expression) (0—no expression, 1 point—>0–10%, 2 points—>10–25%, 3 points—>25–50%, 4 points—>50%) [5,41]. To estimate the cytoplasmic expression levels of SLUG, TWIST, SNAIL, FNDC5/Ir, and N-cadherin, we used the semiquantitative method immunoreactive score (IRS) according to Remmele and Stegner [42]. The final result of the estimation was the outcome of the multiplication of the obtained points from the intensity of the color reaction (1—weak, 2—moderate, 3—strong) and the percentage of IRS-positive cancer cells (0 point—lack of expression, 1 point—>1–10%, 2 points—>10–50%, 3 points—>50–80%, 4 points—>80%). The expression of the E-cadherin antigen was evaluated quantitatively by estimating the percentage of positive tumor cells (0–5% = no reaction (0 points), 6–25% = weak reaction (1 point), 26–50% = moderate reaction (2 points), above 50% = intense reaction (3 points)).

### 4.4. Real-Time PCR (RT-PCR) Analysis

RT-PCR reactions were performed for BC cell lines and the control cell line. The RNeasy Mini Kit (Qiagen) was used for RNA isolation. The High-Capacity cDNA Reverse Transcription Kit (Applied Biosystems, Waltham, MA, USA) with RNase Inhibitor (Applied Biosystems) was used to perform the reverse transcription reaction. The 7900HT Fast Real-Time PCR System (Applied Biosystems) and the relative quantification (RQ) method were used to analyze mRNA *FNDC5* expression (FNDC5; TaqMan Gene Expression Assay, Applied Biosystems) in the cell lines. The RQ Manager 1.2 software (Applied Biosystems) was used for the analysis. The results were standardized by the reference gene for β-actin expression (ACTB; TaqMan Gene Expression Assay, Applied Biosystems). Changes in the mRNA *FNDC5* level in BC cells were assessed in relation to normal cells. RT-PCR was repeated three times.

### 4.5. Immunofluorescence (IF)

For 24-h microculture, 600 μL of 20 × 10^4^ cells/mL suspension of cells was instilled into each well of Millicell EZ 8-well glass slides (Merck). Microcultures with cells were incubated at 37 °C for 24 h. Then, the cells were fixed using 4% formaldehyde. Subsequently, the fixed cells were incubated with the specific polyclonal rabbit anti-irisin/FNDC5 antibody (dilution 1:50; code no. NBP2-14024; Novus Biologicals) at 4 °C overnight. The slides with the fixed cells were rinsed and incubated for 1 h with donkey anti-rabbit secondary AlexaFluor 568 conjugated antibody (dilution 1:2000; code no. A10042; Invitrogen, Waltham, MA, USA). The secondary antibody was diluted in a background-reducing reagent (Agilent Technologies, Santa Clara, CA, USA). The Prolong DAPI Mounting Medium (Invitrogen) was used to stain the cell nucleus and mount the slides. The observations were made at ×600 magnification using Olympus Fluoview FV3000 confocal microscopy coupled with CellSense (Olympus) software.

### 4.6. Enzyme-Linked Immunosorbent Measurement (ELISA) Tests

The enzyme-linked immunosorbent assay (ELISA) tests were performed on serum samples from BC patients. Serum samples were obtained from venous blood after centrifugation at 3000 rpm for 10 min and were preserved at −80 °C for further analysis. Serum Ir levels were examined using commercially available ELISA recombinant irisin kit test Irisin, Recombinant Elisa kit (Cat No. EK-0670-29, Phoenix Pharmaceuticals, CA, USA). The ELISA tests were performed according to the manufacturer’s protocol. For each sample, 50 μL of serum was used. The results were verified by microplate reader ELX-800 (BIO-TEK, VT, USA). The examination was performed in two repetitions.

### 4.7. Transmission Electron Microscopy (TEM)

After the application of TEM, the subcellular localization of Ir was examined in two types of samples. Breast tumors sections were cut into small pieces and in vitro culture of the MDA-MB-468 cell line representing metastatic adenocarcinoma of the breast was immersed in a 4% solution of PBS and paraformaldehyde (Boster Bio, Pleasanton, CA, USA) and fixed overnight at 4 °C (for tumors) or 25 min at room temperature (for the cell line). Subsequently, the fixed cells were gently removed with a cell scraper and placed in conical tubes (15 mL). After three cycles of centrifugation (1800 rpm for 8 min), the fixative was washed with PBS and distilled water. The cell pellets were entrapped in a fibrin clot formed from bovine thrombin (lyophilizate reconstituted with PBS; Biomed, Lublin, Poland) and fibrinogen (1 mg/mL; Merck KGaA, Darmstadt, Germany). To preserve cellular ultrastructure, each sample was post-fixed for 7 min in a 0.25% (*w*/*v*) solution of PBS and osmium tetroxide (Serva Electrophoresis, Heidelberg, Germany). Following washing with PBS (3 × 5 min), dehydration of the specimens was completed in a graded series of ethanol (2 × 50%, 2 × 70%, 1 × 96%, 4 × 100%; Stanlab, Lublin, Poland). The samples were incubated with a mixture of 99.8% ethanol and LR White resin (medium catalyzed) (Polysciences, Inc., Warrington, PA, USA) combined in appropriate proportions, i.e., 2:1 (20 min), 1:1 (1 h), and 1:2 (1 h), respectively. Next, the material labeled with the corresponding number was placed at the bottom of the gelatine capsule (flat embedding molds; Pelco, Ted Pella, Redding, CA, USA), embedded in pure resin, and underwent the hardening process in the laboratory incubator (55 °C for 48 h). Resin blocks were sectioned using the RMC ultramicrotome (Power Tome XL; Tucson, AZ, USA) into 700-nm-thick semithin sections. These sections, which were mounted onto the slide on the heating plate (at 100 °C), were stained with 1% aqueous toluidine blue (Serva Electrophoresis, Heidelberg, Germany) mixed with sodium carbonate (Alchem, Toruń, Poland). This staining is dedicated to semithin sections and allows obtaining different intensities of tissue fragments, which facilitates trimming of the area, cancer cells, and stroma with the exclusion of fat tissue. After trimming, serial 80-nm-thick ultrathin sections were obtained using the ultra-diamond knife and placed on the grids (nickel-made; Ted Pella, Redding, CA, USA). The immunogold reaction was performed to trace the distribution of Ir. All steps were performed in the Petri dish. The first step was based on blocking residual aldehyde groups by placing the sections in 0.02 M glycine solution (BioShop Canada Inc., Burlington, ON, Canada) in PBS for 10 min. Next, the samples were immersed in triton X-100 (0.1% in PBS; Bioshop, Burlington, ON, Canada) to increase membrane permeability and then washed with PBS (5 min, 3 times). The sections were subsequently blocked for 1 h in a 1% bovine serum albumin (BSA; Carl Roth, Mannheim, Germany) to inhibit the nonspecific antigen-binding sites and quickly washed with PBS.

Next, ultrathin sections were incubated in the appropriate antibodies diluted in 0.1% BSA in PBS—primary polyclonal antibody against Ir (1:10 dilution, code no. NBP2-14024; Novus Biologicals, Littleton, CO, USA) and then anti-rabbit secondary antibody tagged with colloidal gold particles (1:10 dilution, code no. ab27237, goat anti-rabbit IgG H&L, 20 nm gold; Abcam, Cambridge, UK) for 1 h in a dark chamber. The steps were as follows: grids were rinsed in PBS and distilled water, post-fixed in 1% glutaraldehyde (Serva Electrophoresis, Heidelberg, Germany), and post-stained with the UranyLess solution and Reynolds lead citrate 3% (Electron Microscopy Sciences, Hatfield, PA, USA) to preserve the ultrastructure quality and washed thoroughly in distilled water. JEM-1011 (Jeol, Tokyo, Japan) transmission electron microscope operating at the accelerating voltage of 80 kV was used. Electronograms were obtained using the TEM imaging platform (iTEM1233) equipped with a Morada Camera (Olympus, Tokyo, Japan).

### 4.8. Statistical Analysis

The distribution of the results was checked by the Kolmogorov–Smirnov test. The differences in the serum levels of markers or tissue expression in BC and NMBD and their association with the clinicopathological factors were analyzed by the Kruskal–Wallis test or the Mann–Whitney U test. Correlations between Ir and E-cadherin, N-cadherin, SNAIL, and SLUG proteins were evaluated by the Spearman rank test. The unpaired t-test was used to assess the differences between the Ir level in BC and the control cell lines. Statistical analysis was performed using Prism 5.0 (GraphPad, La Jolla, CA, USA). P values below 0.05 were considered statistically significant.

## Figures and Tables

**Table 1:** Characteristics of BC patients related to low and high expression of Ir (divided according to the median) compared with the Chi^2^ test (from Cebulski et al., 2022 [2]).

Clinicopathological Parameter	*n* 541 (%)	Irisin Expression in BC Cells		
		Low >0–5.6	High ≥5.6–12	Chi^2^ Test *p*
**Age**				0.0707
**≤ 60**	156 (28.8)	75 (48.1)	81 (51.9)	
**>60**	385 (71.2)	218 (56.6)	167 (43.4)	
**Histological type**				0.7252
**IDC**	521 (96.3)	281 (53.9)	240 (46.1)	
**ILC**	12 (2.2)	7 (58.3)	5 (41.7)	
**IPC**	1 (0.2)	1 (100.0)	0 (0.0)	
**MC**	1 (0.2)	0 (0.0)	1 (100.0)	
**MetC**	4 (0.7)	3 (75.0)	1 (0.0)	
**MucC**	2 (0.4)	1 (50.0)	1 (50.0)	
**Tumor size (T)**				**0.0414**
**T1**	344 (63.6)	183 (53.2)	161 (46.8)	
**[T1a-b**	[89 (16.5)	35 (39.3)	54 (60.7)	
**T1c]**	255 (47.1)]	148 (58.0)	107 (42.0)	
**T2**	179 (33.1)	99 (55.3)	80 (44.7)	
**T3-4**	18 (3.3)	11 (61.1)	7 (38.9)	
**Lymph nodes (N)**				**0.0258**
**N0**	340 (62.8)	169 (49.7)	171 (50.3)	
**N1**	193 (35.7)	119 (61.7)	74 (38.3)	
**N2**	8 (1.5)	5 (62.5)	3 (37.5)	
**Stage**				0.5507
**I**	237 (43.8)	123 (51.9)	114 (48.1)	
**II**	283 (52.3)	157 (55.5)	126 (44.5)	
**III**	21 (3.9)	13 (61.9)	8 (38.1)	
**Histological grade (G)**				0.2929
**G1**	96 (17.7)	49 (51.0)	47 (49.0)	
**G2**	350 (64.7)	198 (56.6)	152 (43.4)	
**G3**	95 (17.6)	46 (48.3)	49 (51.7)	

BC—breast cancer; IDC—invasive ductal carcinoma, ILC—invasive lobular carcinoma, IPC—invasive papillary carcinoma, MC—medullary carcinoma, MetC—metaplastic carcinoma, MucC—mucinous carcinoma.

**Table 2:** Characteristics of BC patients related to low and high serum Ir levels (divided according to the median) compared with the Chi^2^ test. Statistically significant results are marked in bold.

Clinicopathological Parameter	*n* 77 (%)	Irisin Serum Concentrations in BC Patients		
		Low <8.9 ng/mL	High ≥8.9 ng/mL	Chi^2^ Test *p*
***Age***				**0.0127**
***≤60***	51 (66.2)	20 (39.2)	31 (60.8)	
***>60***	26 (33.8)	18 (69.2)	8 (30.8)	
**Tumor size (T)**				0.1816
**T1**	41 (53.2)	18 (43.9)	23 (56.1)	
**T2**	27 (35.1)	17 (63.0)	10 (37.0)	
**T3-T4**	9 (11.7)	3 (33.3)	6 (66.7)	
**Lymph nodes (N)**				0.6469
**N0**	36 (46.8)	17 (47.2)	19 (52.8)	
**N1**	33 (42.9)	18 (53.3)	15 (46.7)	
**N2**	8 (10.4)	3 (23.1)	5 (76.9)	
**Histological grade (G)**				0.1194
**G1**	19 (24.7)	14 (73.7)	5 (26.3)	
**G2**	49 (63.6)	23 (46.9)	26 (53.1)	
**G3**	9 (11.7)	4 (44.4)	5 (55.6)	

**Table 3:** Associations of Ir serum level with the clinicopathological characteristics in patients with BC. Statistically significant results are marked in bold.

Comparison of BC Groups	*p* (Mann–Whitney U Test)	BC Groups	Mean Value ± SD
Tumor size (T)		Tumor size (T)	
**T1 vs. T2**	0.6348	**T1**	12.50 ± 13.3
**T1 vs. T3-4**	0.2273	**T2**	9.50 ± 2.4
**T2 vs. T3-4**	0.2618	**T3-T4**	12.85 ± 6.3
**Lymph nodes (N)**		**Lymph nodes (N)**	
**N0 vs. N1**	0.4937	**N0**	9.25 ± 2.1
**N0 vs. N2**	**0.0055**	**N1**	10.33 ± 7.7
**N1 vs. N2**	**0.0072**	**N2**	20.86 ± 23.7
**Histological grade (G)**		**Histological grade (G)**	
**G1 vs. G2**	0.3224	**G1**	8.02 ± 1.4
**G1 vs. G3**	**0.0166**	**G2**	8.96 ± 2.2
**G2 vs. G3**	0.2268	**G3**	9.86 ± 1.4

## Data Availability

The raw data and the analytic methods will be made available to other researchers for the purposes of reproducing the results in their own laboratories upon reasonable request. To access protocols or datasets contact katarzyna.nowinska@umw.edu.pl.
